# The current treatment landscape in the UK for stage III NSCLC

**DOI:** 10.1038/s41416-020-01069-z

**Published:** 2020-12-08

**Authors:** Matthew Evison, on behalf of AstraZeneca UK Limited

**Affiliations:** North West Lung Centre Wythenshawe Hospital, Manchester University NHS Foundation Trust, Manchester, UK

## Abstract

For stage III non-small cell lung cancer (NSCLC), approximately a third of patients survive up to 5 years, with decreasing 5-year survival rates for stage IIIB and stage IIIC disease. Although curable, stage III NSCLC encompasses a diverse range of disease presentation, with an equally complex range of multi-modal treatment options, including systemic and local therapies for distant and local disease control, respectively. This complexity results in a number of challenges for the multi-disciplinary team (MDT) in achieving optimal treatment outcomes for patients. As multi-modality treatment is the preferred treatment strategy for all stage III disease, the focus of this article is the key surgical, chemotherapy and radiotherapy clinical trials as well as guidelines that currently outline radical therapy options for patients with both potentially resectable and unresectable stage III NSCLC.

## Background

Stage III non-small cell lung cancer (NSCLC) describes ‘locally advanced’ lung cancer where there are adverse prognostic features within the primary tumour (based on size, distribution or relationship to adjacent structures) and/or the presence of metastatic disease only within regional lymph nodes. A significant proportion of stage III NSCLC is made up of patients with N2 disease, where the primary tumour has metastasised to the ipsilateral mediastinal lymph nodes. Despite this burden of local disease, there are no distant metastases in stage III NSCLC and therefore treatment with radical intent can be considered. Stage III NSCLC is highly heterogeneous with a wide spectrum of disease distribution and an equally complex range of treatment options. In general, the optimal treatment regimen is multi-modal with systemic and local therapies for distant and local disease control, respectively. The exact sequence and modality used is keenly debated and highly case specific. Although treatment with radical intent is recommended in stage III NSCLC, the outcomes remain poor with only a small fraction of patients achieving a long-term response. In studies that evaluated multi-modality treatment in patients with unresectable stage III NSCLC and a good performance status (PS; defined as an Eastern Cooperative Oncology Group PS 0–1), utilising modern day staging and treatment techniques, the 5-year overall survival (OS) rate was 20%.^1^ New treatment regimens and techniques as well as fair and equal access to optimal treatment and expert teams, to minimise the risks and complications from treatment and to standardised practice, are key goals in addressing this critical issue.

## Eighth edition of the tumour, node and metastases (TNM) classification of lung cancer: stage III NSCLC

On January 1, 2018, the United Kingdom (UK) adopted the eighth edition of the TNM lung cancer staging system proposed by the International Association for the Study of Lung Cancer (IASLC) and accepted by the Union for International Cancer Control and the American Joint Committee on Cancer^2–5^. The eighth edition is now widely used across Europe and the updated TNM staging system has a number of changes in relation to stage III lung cancer compared with the seventh edition (Tables 1 and 2). First, the classification of T3 and T4 tumours has changed. In the eighth edition, T3 includes primary tumours measuring >5 cm but ≤7 cm and T4 tumours are those measuring >7 cm, whereas in the seventh edition, only tumours measuring >7 cm were classified as T3 and only tumours invading major structures were classified as T4. Tumours that were previously classified as T3 in the seventh edition, because of their location in the main bronchi being <2 cm from the carina or because of atelectasis of the entire lung, have been re-classified as T2 tumours in the updated version (Table 1). There are also a number of changes within the overall stage groupings of stage III NSCLC (Table 2). T3 N2 M0 has changed from stage IIIA in the seventh edition to stage IIIB in the eighth edition. There is also a new stage of IIIC, which incorporates T3–4 N3 M0, previously classified as stage IIIB. Therefore, the three-stage groupings that make up stage III NSCLC in the eighth edition TNM are IIIA, IIIB and IIIC and the 5-year survival for these groups, based on the IASLC pathological staging database analysis, were 36, 26 and 13%, respectively.^5^ It is important to note that the IASLC also depict OS by clinical staging, which is often seen as inferior to pathological staging.^6,7^ In particular, radiological modalities do not provide the required sensitivity to accurately stage the mediastinum and there is a greater emphasis on the importance of high-quality pathological nodal staging in the diagnostic work-up of stage III lung cancer. In one recent study, clinical staging misclassified the nodal staging in 38% of cases when compared to the final pathological staging from surgical resection.^7^

**Table 1:** Differences in stage III NSCLC tumour stage classification criteria between the seventh and eighth edition of the tumour, node and metastases classification of lung cancer.

Stage	Seventh edition TNM	Eighth edition TNM
T3	A primary tumour >7 cm Or a tumour that invades: • Parietal pleura • Chest wall • Phrenic nerve • Diaphragm • Mediastinal pleura • Pericardium Or a tumour <2 cm from the carina in the main bronchi Or a tumour causing atelectasis/obstructive pneumonitis of the entire lung Or a tumour with a separate tumour nodule in the same lobe	A primary tumour >5 cm but ≤7 cm Or a tumour that invades: • Parietal pleura • Chest wall • Phrenic nerve • Pericardium Or a tumour with a separate tumour nodule in the same lobe
T4	A tumour that invades: • Mediastinum • Heart • Great vessels • Trachea • Recurrent laryngeal nerve • Oesophagus • Vertebral body • Carina Or a tumour with a separate tumour nodule in a different ipsilateral lobe	A primary tumour >7 cm Or a primary tumour that invades: • Diaphragm • Mediastinum • Heart • Great vessels • Trachea • Recurrent laryngeal nerve • Oesophagus • Vertebral body • Carina Or a tumour with a separate tumour nodule in a different ipsilateral lobe

This table was created by the author using guidance from refs. ^44,45^

**Table 2:** Differences in stage III NSCLC tumour stage classification between the seventh and eighth edition of the tumour, node and metastases classification of lung cancer.

Stage	Seventh edition TNM	Eighth edition TNM
T3 N1 M0	IIIA	IIIA
T4 N0 M0	IIIA	IIIA
T4 N1 M0	IIIA	IIIA
T1 N2 M0	IIIA	IIIA
T2 N2 M0	IIIA	IIIA
T3 N2 M0	IIIA	IIIB
T4 N2 M0	IIIB	IIIB
T1 N3 M0	IIIB	IIIB
T2 N3 M0	IIIB	IIIB
T3 N3 M0	IIIB	IIIC
T4 N3 M0	IIIB	IIIC

This table was created by the author using guidance from refs. ^5,44,45^

## Epidemiology and treatment patterns of stage III NSCLC in the UK and Europe

**Fig. 1:**
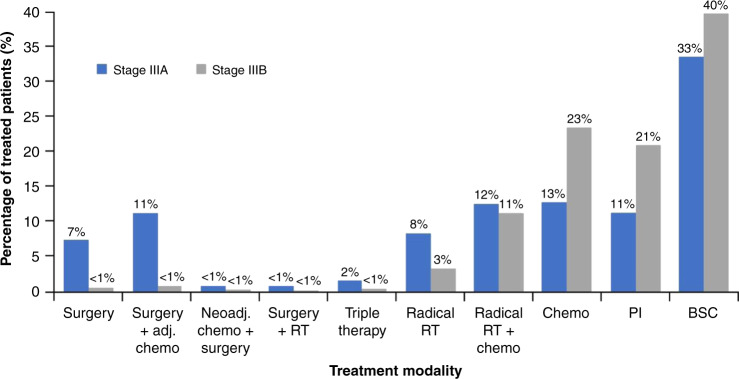
2017 treatment of stage IIIA and IIIB NSCLC in the UK. This figure was created by the author from data published in the UK NLCA annual report 2018.^8^ adj adjuvant, BSC best supportive care, chemo chemotherapy, PI palliative intent, RT radiotherapy.

The 2018 National Lung Cancer Audit (NLCA) report presented data from 39,205 cases of primary lung cancer diagnosed in the UK between January 1 and December 31, 2017.^8^ Overall, 20% of primary lung cancer cases in this audit were stage III, with 11% classified as stage IIIA and 9% as stage IIIB. The 1-year survival rate for patients with stage III NSCLC in a previous 2017 audit was 42.5% (the current audit does not separate survival rates by stage),^9^ while the 5-year survival rate for these patients in the UK has been published at 6%.^10^ For stage IIIA, in 2017, 44.8% of patients received palliative therapy or best supportive care and 25.2% received bimodality treatment with surgery and chemotherapy, surgery and radiotherapy or radiotherapy and chemotherapy. For stage IIIB, 60.4% of patients received palliative therapy or best supportive care and 12.1% received bimodality treatment with surgery and chemotherapy, surgery and radiotherapy, or radiotherapy and chemotherapy (Fig. 1). While the management of stage III NSCLC is a rapidly evolving area with new excitement on the potential impact of immunotherapy, the reality is that the majority of UK patients do not receive radical intent treatment. Reasons for this are likely to be multi-factorial, perhaps reflecting impaired physiological reserve in lung cancer patients or variability in clinical practice and expertise across the UK. However, the NLCA data above should act as a driver for the review of international and local treatment rates and, where needed, improve the radical intent treatment rates for patients with stage III NSCLC. Accelerated pathways, high-quality diagnostic and staging procedures, prehabilitation, smoking cessation, nutritional support and standardised multi-disciplinary team (MDT) protocols are all important strategies to help address these outcomes.

In the context of Europe, an overall real-world analysis investigated the treatment management of patients with NSCLC from eight European countries, including 3508 patients from 182 sites.^11^ For both stage IIIA and IIIB disease, a total of 22.3% received surgery with the addition of neoadjuvant chemotherapy, adjuvant chemotherapy, radiotherapy or chemoradiotherapy (CRT). Of the 77.7% of patients who did not have surgery, 54.9% received treatment with radiotherapy, 43.8% of which was in the neoadjuvant setting and 42.1% of patients received concurrent chemotherapy with 3.3% in the adjuvant setting. The analysis only reported 1-year survival rates, which were 79% for stage IIIA and 61% for stage IIIB disease. In addition, it is not clear whether survival based on treatment is related to NSCLC histology, with sources providing conflicting data on outcomes for patients with adenocarcinoma or squamous cell stage III disease.^12,13^ The dynamics of recurrence after multi-modality therapy for stage III NSCLC are also a confounding factor for survival, as they are organ specific and vary according to pathologic factors.^14^

## Deciding on optimal treatment regimens for stage III NSCLC

### Definition of resectable stage III NSCLC

The first and most imperative question in the management of stage III NSCLC, assuming adequate physiological reserve to withstand optimal treatment, is whether the disease is potentially resectable. The answer to this question defines whether surgery might have a role in multi-modality treatment. One issue embedded in the management of stage III NSCLC is the lack of an agreed definition of ‘potentially resectable’.^15^ This will inherently lead to heterogeneity in the patient population with potentially resectable stage III NSCLC, as well as difficulties comparing and pooling data from different sources. Combining the information described within international guidelines on stage III NSCLC, a suggested definition of potentially resectable stage III disease has been proposed, which centres on the requirement for systematic clinical and pathological staging and a high probability of complete resection with clear pathological margins. The author’s proposed definition, specifically for N2 disease, is provided in Table 3. Beyond N2, N3 disease is considered unresectable, while patients with a tumour defined as T4 fall into a heterogeneous group, with some being unresectable based on either the size or the invasiveness of the tumour. Inevitably, the decision will come down to the surgical opinion provided within the MDT meeting.

**Table 3:** Proposed definition of ‘potentially resectable stage III NSCLC’.

• Pathologically confirmed NSCLC
• Thorough pathological nodal staging completed (surgical or endoscopic)
• Thorough radiological staging including at least PET-CT and MRI brain with contrast
• Primary tumour resectable with high probability of clear pathological margins and complete resection
• Any nodal disease is discrete, easily measurable and defined, free from major mediastinal structures including the great vessels and trachea with no individual lymph node measuring >3 cm

This table was created by the author using guidance from ref. ^46^

MRI magnetic resonance imaging, NSCLC non-small cell lung cancer, PET-CT positron emission tomography–computed tomography.

### Reviewing treatment regimens for unresectable stage III NSCLC

There is little debate as to the optimal treatment regime in unresectable stage III NSCLC, with definitive CRT being the standard of care. Concurrent chemoradiotherapy (cCRT) is preferred over sequential CRT (sCRT) assuming patients have adequate physiological reserve, with cisplatin-based chemotherapy preferred over carboplatin for cCRT because of the survival advantage demonstrated in clinical trials.^16–18^ A meta-analysis performed by the NSCLC Collaborative Group of six trials and 1205 patients evaluated cCRT versus sCRT in patients with stage III NSCLC and reported an absolute survival benefit of 5.7% at 3 years and 4.5% at 5 years, in favour of the concurrent approach.^19^ In a large randomised controlled trial (*n* = 610) by the Radiation Therapy Oncology Group (RTOG 9410), cCRT was superior to sCRT with an increase in median OS from 14.6 months to 17 months and a significant increase in the 5-year survival rate from 10% to 16%, respectively, (*P* = 0.046).^20^ For unresectable stage III NSCLC, if a patient has significantly impaired physiological reserve, they would likely be referred for palliative treatment over intensive multi-modal treatment. However, in stage III NSCLC where the disease is potentially resectable, there is significant debate as to the optimal treatment. There are four key randomised controlled trials that have addressed this challenging cohort of patients, the majority of which have focussed on N2 NSCLC where the greatest debate lies. These key trials are reviewed in subsequent sections.

### CRT, chemosurgery and trimodality treatment in stage IIIA-N2 NSCLC

It has been well established in historic trials that the combination of systemic therapy plus a local therapy is superior to a local therapy alone. For example, the importance of chemotherapy in combination with surgery was outlined in a randomised trial comparing preoperative chemotherapy plus surgery with surgery alone in patients (*n* = 60) with stage IIIA NSCLC.^21^ The median OS was 26 months in patients treated with both chemotherapy and surgery compared with 8 months in the surgery alone group (*p* < 0.001). The debate, however, rests with which form of multi-modality treatment is superior to another. The EORTC 08941 study recruited patients from 1994 to 2002,^22^ which covers a period prior to the routine use of positron emission tomography (PET) imaging and endobronchial ultrasound (EBUS) nodal staging in clinical practice. Patients (*n* = 332) with unresectable stage IIIA-N2 NSCLC who had a disease response following three cycles of induction chemotherapy were randomised to either radical radiotherapy or surgical resection. No significant difference in median progression-free survival (PFS; 9 versus 11.3 months, respectively; hazard ratio (HR) 1.06, 95% confidence interval (CI) 0.85–1.33; *P* = 0.6) or OS (5-year OS: 15.7 versus 14%; HR 1.06, 95% CI 0.84–1.35; *p* = 0.596) was observed. Acute grade 3 or 4 oesophageal adverse events (AEs) were observed in 1 patient (<1%) and grade 3 or 4 pulmonary events in 5 patients (4%). Late pulmonary and oesophageal AEs were reported in 11 (7%) and 1 (<1%) patients, respectively, with 1 death from radiation pneumonitis. Although this trial compared the two most commonly used bimodality treatment strategies in the UK, it found no superior treatment for high-volume stage IIIA-N2 NSCLC. A few points to note are the multiple types of chemotherapy regimens (including several that were not cisplatin based) and the changes in supportive measures at the time of this study compared with today’s standards. Survival in this study may have also been related to the fact that 47% of patients required pneumonectomy. Increasing the complexity of the surgical intervention is typically associated with an increased risk of postoperative mortality. Therefore, where possible lobectomy is the preferred surgical technique.^23,24^ It is also important to note that, in the EORTC 08941 trial, CRT was delivered sequentially rather than the current standard of care that is cCRT and that chemotherapy was given neoadjuvantly, whereas in the UK, upfront surgery followed by adjuvant chemotherapy is more widely practised.

The Intergroup 0139 trial compared pre-operative CRT followed by surgical resection versus definitive CRT in patients with low-volume mediastinal T1–3 N2 NSCLC.^25^ Between 1994 and 2001, 429 patients were recruited to the study. There was a statistically significant difference in PFS in the trimodality arm (12.8 versus 10.5 months; *p* = 0.017), though this did not translate into a difference in 5-year survival (27% versus 20%; odds ratio 0.63, 95% CI 0.36–1.10; *p* = 0.10). This is likely due to the improved treatment effect of CRT followed by surgery being offset by a high mortality rate in patients undergoing pneumonectomy (26%). A post hoc matched analysis of patients undergoing induction CRT followed by lobectomy versus definitive CRT showed improved survival in the lobectomy group (33.6 versus 21.7 months; *p* = 0.002). Concern has been expressed about the operative mortality in the pneumonectomy group from low-volume centres of the Intergroup 0139 trial, particularly as significantly better operative mortality was reported from high-volume expert centres.^26^ The most common grade 3 or 4 AE in the Intergroup 0139 trial was leukopenia, which occurred in 97 (48%) patients receiving trimodality treatment; other grade 3 or 4 events experienced in this arm included oesophagitis in 20 patients (9.9%) and pneumonitis or other respiratory complications, which occurred in 18 (8.9%) patients. There were no treatment-related deaths during induction cCRT in either treatment arm. Similar treatment modalities have been evaluated in the more recent ESPATUE trial, which found no significant OS difference between patients undergoing induction chemotherapy followed by induction CRT than surgery versus induction chemotherapy followed by definitive CRT.^27^ The SAKK trial found no difference in event-free survival and OS when comparing surgery following either induction CRT or induction chemotherapy in patients with resectable disease.^28^ However, the aforementioned trials are hard to compare as all included slightly different patient populations, with the SAKK and Intergroup 0139 trials including a more ‘positive’ selection of patients with more minimal, less bulky N2 disease, while the ESPATUE trial included more patients with N3 and T4 disease.^25,27,28^

Given the lack of superiority of one treatment regime over another in resectable stage III NSCLC, there have been a number of meta-analyses combining data from randomised controlled trials, which also failed to show superiority of one treatment.^29^ One meta-analysis of trimodality treatment with surgery versus definitive CRT (concurrent or sequential not specified) showed a trend towards a survival benefit with trimodality treatment (HR 0.87; 95% CI 0.75–1.01; *p* = 0.068).^30^

## Guideline recommendations for stage III NSCLC

There are a number of local and international guidelines providing recommendations on the treatment of stage III NSCLC.^15,24,31^ There are some areas of consensus opinion including recommending definitive CRT in unresectable stage III NSCLC and other areas with less consensus. Most guidelines acknowledge that, in resectable stage III NSCLC, pre-operative CRT followed by surgical resection, pre-operative chemotherapy followed by surgery or definitive CRT are possible treatment regimens, none of which have been shown to be vastly superior to another. The 2010 British Thoracic Society (BTS) and Society for Cardiothoracic Surgery Guidelines (SCTS) on the Radical Management of Lung Cancer specifically recommend considering surgery in cases of single-station N2 NSCLC.^31^ This recommendation was heavily influenced by data from the IASLC staging databases, which looked at large populations of patients undergoing surgical resection of lung cancer and systematic lymph node sampling. The results revealed that patients with pathologically staged single-station N2 NSCLC had similar survival rates as those with multi-station N1 disease (5-year survival 35%) and improved survival than those with multi-station N2 disease (5-year survival 20%).^31,32^ This has led some to conclude that single-station N2 NSCLC should be considered a surgical disease as multi-station N1 disease would be. However, while multi-station N2 is a prognostic factor conferring a worse prognosis compared with single-station N2 NSCLC, it is not a predictive factor as there was no comparator group undergoing non-surgical management in the IASLC databases to demonstrate any differences in outcomes.^32^ The randomised controlled trials in resectable stage III NSCLC described within this paper have failed to show vast superiority of multi-modality treatment regimens involving surgery versus those without surgery, both in low- and high-volume mediastinal disease.^27^ This view is supported by the American College of Chest Physician Guidelines for stage III NSCLC which note that the evidence does not support the concept that surgery can only be justified in patients with minimal N2 disease.^15^ A summary of guideline recommendations for stage III NSCLC is provided in Table 4.

**Table 4:** Summary of UK, European and American guidelines on the management of potentially resectable N2 NSCLC.

Guideline	Definition of ‘resectable’	Recommendations	Notes
BTS and SCTS (2010)	Non-fixed lymph nodes Non-bulky lymph nodes Single-zone N2 disease Reasonable chance of: Complete resection Clear pathological margins	Consider surgery as part of multi-modality treatment in non-fixed, non-bulky, single-zone N2 NSCLC Further research into the role of surgery in non-fixed, non-bulky, multi-zone N2 NSCLC	Significant weight placed on IASLC staging database outcomes despite lack of comparator group and lack of clinical N2 Guidelines consider evidence for adjuvant chemotherapy more robust than pre-operative chemotherapy
ACCP (2013)	Discrete lymph nodes Easily measurable and defined lymph nodes Free from major structures, such as the great vessels and trachea	Definitive CRT or induction therapy (chemotherapy or CRT) followed by surgery Surgery followed by adjuvant chemotherapy not recommended	Does not support the concept that surgery can only be justified in patients with minimal N2 disease Pre-operative chemotherapy better than surgery alone in all NSCLC (small studies) and therefore surgery plus adjuvant chemotherapy is not recommended
ESMO (2015)	Minimal, non-bulky N2 disease Single-station N2 disease	Definitive CRT, induction chemotherapy followed by surgery or induction CRT followed by surgery	Paramount importance of an experienced and high-volume multi-disciplinary team (MDT) and treatment centres able to minimise risk and complications from multi-modality treatment highlighted
NCCN (2018)	Low-volume lymph nodes Non-invasive lymph nodes Pathologically proven Measuring <3 cm	Definitive CRT or induction chemotherapy followed by surgery or induction CRT followed by surgery Maintenance durvalumab following cCRT	Benefit from pre-operative chemotherapy is similar to that of post-operative chemotherapy and either approach is justified
NICE (2019)	None provided	Consider CRT followed by surgery	CRT followed by surgery improves PFS and might improve survival compared with CRT alone

This table was created by the author using guidance from refs. ^15,23,24,31,34^

*ACCP* American College of Chest Physicians, *BTS* British Thoracic Society, *CRT* chemoradiotherapy, *cCRT* concurrent chemoradiotherapy, *ESMO* European Society of Medical Oncology, *IASLC* International Association for the Study of Lung Cancer, *NICE* National Institute for Health and Care Excellence, *NCCN* National Comprehensive Cancer Network, *NSCLC* non-small cell lung cancer, *PFS* progression-free survival, *SCTS* The Society for Cardiothoracic Surgery in Great Britain and Ireland.

In 2019, the NICE Lung Cancer Diagnosis and Management Guideline Group undertook a network analysis comparing CRT, chemosurgery and CRT plus surgery as part of an evidence review in the management of stage III-N2 NSCLC.^23,33^ This meta-analysis could not distinguish the odds of survival across the interventions at 4 and 5 years; however, there was a strong (although statistically not significant) trend towards improved survival with CRT plus surgery.^33^ CRT plus surgery was associated with a longer PFS at 4 and 5 years compared with CRT or chemosurgery. In addition, there were less grade 3 or greater AEs with CRT plus surgery than with CRT or chemosurgery. The NICE Guideline Group also developed a cost-effectiveness model and concluded that CRT was more cost effective than chemosurgery (incremental cost-effectiveness ratio (ICER) £52,400/quality-adjusted life year (QALY)) and CRT plus surgery was more cost effective than CRT (ICER £16,900/QALY). Probabilistic sensitivity analysis showed that CRT plus surgery produced more QALYs than CRT and chemosurgery in 97% and 87% of interactions, respectively.^33^ The NICE Guideline Group therefore recommend that patients with stage III-N2 NSCLC who are suitable for surgery are considered for CRT followed by surgery.^23^

Challenges to the NICE guideline recommendations include that the evidence is based on historic trial data, much before the era of PET imaging and EBUS staging, and the guidance fails to consider modern day radiotherapy and surgical techniques. The change to practice that increased use of CRT plus surgery would represent in the UK (currently ~1% of patients with stage III NSCLC receive trimodality treatment^8^) should also be acknowledged. Finally, how immunotherapy impacts on resectable stage III NSCLC, now and in the future, is still under debate and undergoing further research.

## Sequencing of treatments in stage III NSCLC

Only one in five patients with stage III NSCLC have radical intent multi-modality therapy in the UK.^24^ Within this group, there are two main regimens of treatment utilised: chemotherapy combined with radiotherapy (the NLCA data set does not distinguish between concurrent and sequential CRT) and surgical resection followed by adjuvant chemotherapy, which together account for 19.6% of all radical treatments received by stage III patients. Pre-operative chemotherapy followed by surgical resection is used in 0.9% of patients and pre-operative CRT followed by surgery in 1.6% of patients. The use of adjuvant chemotherapy following surgical resection is somewhat at odds with the trial protocols described in this paper, which have universally used pre-operative therapy,^25,28–30^ and the recommendations within the majority of international guidelines are based on these trials.^15,24,31,34^ The question of whether chemotherapy should be given in the pre-operative setting or as adjuvant treatment in the post-operative setting has been reviewed in many of the guidelines detailed here, however, only in the context of all cases of NSCLC surgery and not specifically for stage III disease. The BTS and SCTS guidelines conclude that the evidence base for adjuvant chemotherapy is more robust than that for the pre-operative setting, including, for example, the Lung Adjuvant Cisplatin Evaluation (LACE) meta-analysis of 5 trials and 4584 patients, which demonstrated an overall increase in 5-year survival of 5.4% (HR 0.83; 95% CI 0.72–0.94) in patients with stage III NSCLC who received adjuvant versus pre-operative chemotherapy.^35^ The National Comprehensive Cancer Network (NCCN) 2018 NSCLC guideline concludes that the HRs from pooled analyses of pre-operative chemotherapy are largely similar to the LACE meta-analysis and that either approach is justified. It seems reasonable to conclude therefore that the timing of chemotherapy, either pre-operatively or adjuvant treatment, is less important than ensuring completion of all the elements of the planned multi-modality regime.^34^ For example, a study looking at compliance rates of planned neoadjuvant and adjuvant chemotherapy in relation to surgery in patients with NSCLC (although the patient population [*n* = 624] was mainly stage I patients, 23.8% of patients were stage IIIA-N2) found that 90% of patients completed both neoadjuvant chemotherapy and surgery, compared with 76.2% who completed both surgery and adjuvant chemotherapy.^36^ The main reason for incomplete treatment was patient choice, therefore understanding why a patient would potentially not want to start treatment versus the benefit that it could provide is important.

## Future treatment for stage III NSCLC

Immunotherapy is causing a paradigm shift in advanced-stage NSCLC, setting a standard of care with the KEYNOTE-024 and KEYNOTE-189 trials.^37,38^ Immunotherapy is also being studied in stage III NSCLC and has the potential to change the standard of care in this setting. The randomised controlled PACIFIC trial compared durvalumab (Imfinzi®▼; AstraZeneca UK Limited) given as consolidation therapy following two or more cycles of platinum-based cCRT in stage III NSCLC versus placebo.^39^ This trial met its co-primary endpoint of PFS with a median PFS of 16.8 months for durvalumab compared with 5.6 months for placebo. Results also showed an improvement in the 12-month PFS rate from 35.3% to 55.9% and 18-month PFS rate from 27.0% to 44.2%, favouring durvalumab. Grade 3 or 4 AEs occurred in 29.9% of patients who were treated with durvalumab and 26.1% of those who received the placebo, of which the most common was pneumonia. The PACIFIC trial also met its second co-primary endpoint of a significant improvement in OS following durvalumab treatment compared with placebo (median not reached and 28.7 months, respectively; HR 0.68; 99.73% CI 0.47–0.997; *p* = 0.0025).^40^ Recently, an updated OS analysis, after a median duration of follow-up of 33.3 months, has been reported. This updated analysis was consistent with that previously reported with a 31% reduction in the risk of death (median not reached with durvalumab versus 29.1 months with placebo; stratified HR 0.69; 95% CI 0.55–0.86), with 12-, 24- and 36-month OS rates all improved with durvalumab compared with placebo (83.1% versus 74.6%, 66.3% versus 55.3% and 57.0% versus 43.5%, respectively).^41^ Based on the PFS results, the NCCN 2018 NSCLC guidelines have recommended durvalumab as maintenance therapy for patients with stage III NSCLC, whose disease has not progressed following the completion of platinum-based cCRT (Table 4).^34^ In addition, there may be an option for neoadjuvant or adjuvant immunotherapy in combination with surgical resection in the future for patients with stage III NSCLC.^42,43^

## Conclusions

Stage III NSCLC is a highly complex and heterogeneous disease resulting in a number of challenges to achieve optimal patient outcomes. Patients with stage III NSCLC must undergo intensive multi-modality treatment, with associated treatment-related risks and AEs, in order to optimise the chances of long-term survival; however, long-term survival outcomes remain poor. For stage III NSCLC that is resectable, no one multi-modality regime has been proven to be vastly superior to another and therefore patient choice, shared decision-making and the expertise of the treating MDT are critical in defining the most appropriate treatment regime for individual cases. In stage III NSCLC that is deemed unresectable, the consensus is that cCRT is the most appropriate treatment. Despite this, only one-fifth of patients with stage III NSCLC undergo multi-modality treatment in the UK. The poor outcomes and lack of access to optimal treatment is a call to arms for UK lung cancer teams to improve patient outcomes through prehabilitation and rehabilitation to ensure the best physiological reserve for multi-modality treatment and to build the expertise of MDTs in delivering complex multi-modality treatments for this challenging disease. Durvalumab is a viable therapy option with the potential to become the standard of care as consolidation therapy for those patients with stage III unresectable NSCLC and programmed death-ligand 1 ≥1% who have completed two cycles of platinum-based cCRT and is a positive advancement in addressing the poor outcomes for patients with stage III NSCLC.

## Data Availability

Not applicable.

## Appendix

PRESCRIBING INFORMATION

IMFINZI^®^ ▼(durvalumab) 50 mg/ml solution for infusion

https://medicines.astrazeneca.co.uk/content/dam/multibrand/uk/en/prescribinginformation/imfinzi-pi.pdf.
